# The Design of PAN-Based Janus Membrane with Adjustable Asymmetric Wettability in Wastewater Purification

**DOI:** 10.3390/ma17020417

**Published:** 2024-01-14

**Authors:** Yuehui Wang, Jun Huang, Ye Zhang, Shiwen Zhang, Lili Li, Xuan Pang

**Affiliations:** 1 Key Laboratory of Automobile Materials, Ministry of Education, College of Materials Science and Engineering, Jilin University, Changchun 130022, China; 15161110960@163.com (Y.W.); 17843334500@163.com (J.H.); 15124698738@163.com (Y.Z.); shiwenuknow@163.com (S.Z.); 2 Key Laboratory of Polymer Ecomaterials, Changchun Institute of Applied Chemistry, Chinese Academy of Sciences, Changchun 130022, China

**Keywords:** Janus composite membrane, PCL, oil/water separation, photocatalysis

## Abstract

In this paper, an environmentally friendly polyacrylonitrile-based (PAN-based) composite membrane with a Janus structure for wastewater treatment was successfully fabricated. To achieve the optimum adsorption of PAN-based Janus composite membrane, the asymmetric wettability was regulated through electrospinning, resulting in TiO_2_ modifying PAN as the hydrophilic substrate layer, and PCL gaining a different thickness as the hydrophobic layer. The prepared Janus composite membrane (PAN/TiO_2_-PCL20) showed excellent oil/water separation performance for diverse surfactant-stabilized oil-in-water emulsions. For n-hexane-in-water emulsion, the permeate flux and separation efficiency reached 1344 L m^−2^ h^−1^ and 99.52%, respectively. Even after 20 cycles of separation, it still had outstanding reusability and the separation efficiency remained above 99.15%. Meanwhile, the PAN/TiO_2_-PCL20 also exhibited an excellent photocatalytic activity, and the removal rate for RhB reached 93.2%. In addition, the research revealed that PAN/TiO_2_-PCL20 possessed good mechanical property and unidirectional water transfer capability. All results indicated that PAN/TiO_2_-PCL20 with photocatalysis and oil/water separation performance could be used for practical complex wastewater purification.

## 1. Introduction

In recent years, water pollution problems from frequent oil spills and discharge of industrial oily wastewater have posed a potential threat to the ecological environment and human health [1,2]. The wastewater containing organic oils and dyes from the process of industrial production needs to be settled urgently due to its toxicity, non-biodegradability, carcinogenicity and mutagenicity [3,4,5,6]. Therefore, it is necessary to develop efficient and environmentally-friendly technology to remove organic contaminants from the water environment. 

Traditional methods for treating oil-contaminated wastewater, such as biological treatment, membrane separation, flocculation, air flotation, gravity separation and adsorption etc., have been reported [7,8,9,10,11,12,13,14,15]. Among them, the membrane separation method attracts tremendous attention in oil/water separation because of its high separation efficiency, wide application range and easy operation [16,17,18]. Han et al. fabricated a titanium dioxide-acrylonitrile-butadiene-styrene composite membrane (TiO_2_-ABS) via 3D printing. This TiO_2_-ABS composite membrane demonstrated exceedingly high flux (1.8 × 10^5^ L m^−2^ h^−1^) and oil rejection rate (99.5%) [19]. 

The conventional membrane separation method for water purification is not efficient enough. Recently, some researchers retained the filtration properties and fabricated membranes used in an integrated filtration-adsorption process [20,21]. Photocatalytic degradation is considered to be an effective and green approach for traditional organic dye adsorption and removal [22,23]. Zhao et al. prepared a n-p type Bi_2_WO_6_/AgInS_2_ S-scheme heterojunction for organic pollutants degradation. The Bi_2_WO_6_/AgInS_2_ exhibited much higher photocatalytic activity, achieving degradation efficiency of 97% for RhB under visible light for 60 min [24]. Recently, the utilizing of both photocatalysis and membrane adsorption technologies have become a research hotpot for attempts to remove organic dyes in wastewater [25,26]. Baig et al. fabricated superhydrophobic/superoleophilic photocatalytic membranes (CeO_2_ nanoparticles coated membrane) that exhibited simultaneously high removal efficiency of dyes (99.96%) and oils (99.95%) [27]. 

The name “Janus membrane” comes from Janus, the two-faced god in ancient Roman mythology [28]. Janus membrane is a general term for membranes with opposite properties, such as composition, morphology, wettability, surface charge, and so on. Layer-by-layer electrospinning, surface coating, unilateral deposition and chemical modification are often used to prepare Janus membranes [29,30,31,32]. In recent years, Janus membranes with a unique hierarchical structure of asymmetric hydrophilicity/hydrophobicity have attracted more and more attention [33,34]. Meanwhile, Janus membranes are widely used as an ideal material for water treatment [35,36]. Chen et al. fabricated a Janus wood (JW) through a unidirectional vacuum impregnation method that had a unidirectional transport of water, and presented high flux (3700 L m^−2^ h^−1^) and separation efficiency (99.6%) [37]. Sun et al. fabricated a Janus membrane via hydrophilic ZnO nanowires modified hydrophobic polyvinylidene fluoride (PVDF) nanofiber. The membrane showed excellent oil/water separation fluxes (1210 L m^−2^ h^−1)^ for heavy oil-water mixtures; 7653 L m^−2^ h^−1^ for light oil-water mixtures; and good photocatalytic degradation performance (95%) [38].

Herin, PAN and PCL were used as a matrix to design a multifunctional oil-water separator with a Janus membrane structure. In order to improve its oil-water separation efficiency and endow it with multifunctionality for water remediation, TiO_2_ was added to modify the hydrophilic PAN layer, which was expected to improve oil-water separation performance by modifying asymmetric wettability, and further endowing the multifunction (such as photocatalytic degradation and unidirectional water transfer capability) of the Janus membranes. The morphology, chemical structure, and wettability of all the as-prepared PAN/TiO_2_-PCLs were characterized by SEM, LSCM, FT-IR and contact angle measurement. The oil/water separation performance of the PAN/TiO_2_-PCLs were operated by a homemade experiment device. The photocatalytic degradation performance of the PAN/TiO_2_-PCL20 was investigated under simulated solar irradiation. The mechanical properties and unidirectional water transfer capability were also tested.

## 2. Materials and Methods

### 2.1. Materials

Polyacrylonitrile (PAN, Mw = 150,000), Polycaprolactone (PCL, Mw = 50,000), TiO_2_ nanoparticles (anatase-type nano powder, particle size: 5~10 nm), dichloromethane, coloring agent: Rhodamine B (RhB), Sudan III and methylene blue were provided by Aladdin Industrial Corporation, China. Sodium dodecyl sulfate (SDS, ACS reagent) was purchased from Saan Chemical Technology (Shanghai) Co., Ltd., China. Chloroform (CF), n-hexane, glycerin and N,N-dimethylformamide (DMF) were obtained from Beijing Chemical Works, Beijing, China. Diesel oil was supplied from National Petroleum Co., Ltd., China. Olive oil was purchased from China Oil & Foodstuffs Corporation (COFCO) (Beijing, China). Benzyl benzoate was obtained from Xilong Chemical Technology Co., Ltd., Guangzhou, China. 1,1,2,2-Tetrabromoethane was purchased from Jiangshun Chemical Technology Co., Ltd., Guangzhou, China. All chemical reagents and drugs used in this study were analytical reagents without further purification.

### 2.2. Fabrication of PAN-Based Janus Membrane

#### Fabrication of PAN/TiO_2_-PCL Janus Membrane

**Scheme 1:**
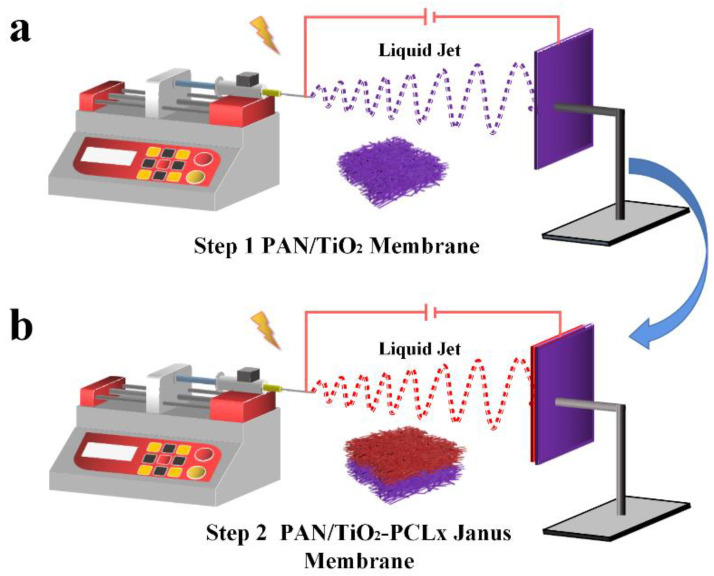
The fabrication process of (**a**) electrospinning PAN/TiO_2_ substrate; (**b**) electrospinning PCL layer on PAN/TiO_2_ substrate.

1 g PAN and 0.3 g TiO_2_ were dissolved in 10 mL DMF by magnetic stirring for 12 h at room temperature, and then PAN/TiO_2_ precursor solution was obtained. At the same time, 2 g PCL was added into 10 mL CF/DMF (4:1, *v*/*v*) mixed solvent to obtain 20 wt% PCL precursor solution. Both precursor solutions were placed into 5 mL syringes. The PAN/TiO_2_ substrate layer was fabricated by electrospinning at 16 kV applied voltage and 1 mL h^−1^ feeding speed for 2 h. Then the PCL fiber membranes with different thickness were electrospun on PAN/TiO_2_ substrate layer with an applied voltage of 12 kV and a flow rate of 2 mL h^−1^ for different electrospinning times (10 min, 20 min and 30 min, respectively). The prepared Janus membranes were named PAN/TiO_2_-PCLx, where x represented the electrospinning time of PCL. The manufacturing process of PAN/TiO_2_-PCLx was schematically displayed in Scheme 1. The PAN-PCLx Janus membranes were prepared by the same procedure and more details were referred to the Supplementary Materials.

### 2.3. Characterizations

The morphologies of all prepared nanofiber membranes were observed by a cold-field emission-scanning electron microscope (SEM, JSM-6700F, JEOL, Tokyo, Japan) at 8 kV acceleration voltage under the vacuum condition. The nanofiber diameters were assisted by Image J software (http://cnij.imjoy.io/, accessed on 17 April 2023). Viscometer (NDJ-1, Shanghai Precision Instruments Corporation, Shanghai, China) was used to measure the viscosity of the solution, and conductivity of the solution was measured by using a conductivity meter (DDS-11A, Shanghai Precision Instruments Corporation, Shanghai, China). The specific surface area and porosity of all prepared membranes were measured according to the Brunauer-Emmett-Teller analyzer (BET, Autosorb-iQ, Quantachrome Instruments, Boynton Beach, FL, USA). The surface morphology and roughness of membranes were detected through a Confocal Laser Scanning Microscope (LSCM, OLS3000, Olympus Corporation, Tokyo, Japan). The chemical compositions and functional groups of all samples were analyzed through a Fourier-transform-infrared spectrometer (FT-IR, FTIR-4100, JASCO, Tokyo, Japan). The mechanical strength was tested on an electronic universal testing machine (XQ-1C, Shanghai New Fiber Instrument Co., Ltd., Shanghai, China). The underwater oil contact angles (UOCAs) were tested by using a droplet shape analyzer (DSA100, Cruise, Berlin, Germany). The droplet size distribution of oil-water emulsion, both before and after filtration, was measured through dynamic light scattering (DLS, Nano ZS90, Malvern Instruments Ltd., Malvern, UK) and optical microscopy (TE2000-U, Nikon, Tokyo, Japan). The photocatalytic performance of the membranes was studied by using an ultraviolet spectrophotometer (UV-6100s, MAPADA, Shanghai, China).

### 2.4. Oil/Water Separation Experiments

#### 2.4.1. Separation of Immiscible Oil-Water Mixtures

The separation experiments of immiscible oil-water mixtures were performed under gravity. The immiscible oil-water mixtures were prepared by mixing water (methylene blue dyed) and oil (1:1, *v*/*v*). The separation effective area was 12.96 cm^2^. During separation, water was collected after penetrating the membrane, while oil was rejected on the membrane. After separation, the device was left to stand for 10 min to ensure that all water droplets permeated through. The separation efficiency (%) was determined by the Equation (1):

(1) $$Efficiency(\%)=(V/V_{i})\times 100$$

where $V_{i}$ (L) and V (L) represented the volume of water before and after oil/water separation, respectively. In addition, the water permeate flux (L m^−2^ h^−1^) was calculated according to the following Equation (2):

(2) $$Flux=\frac{V}{S\times ∆t}$$

where V (L) was the volume of water permeated through the membrane, S (m^2^) was the effective filtration area of membrane, and ∆t (h) was the total filtration time.

#### 2.4.2. Separation of Oil-in-Water Emulsions

For the preparation of the oil-in-water emulsions, SDS was chosen as the emulsifier. A total of 5 mL oil and 500 mL water were taken into a beaker, and then added 0.1 g Span 80 at 1200 rpm for 24 h by stirring. Ultimately, the surfactant-stabilized emulsion was obtained. N-hexane, diesel oil, olive oil, benzyl benzoate, 1,1,2,2-tetrabromoethane and glycerol were used as oils. The separation efficiencies and permeate fluxes were calculated by Equations (1) and (2), respectively. 

The reusability of the PAN/TiO_2_-PCL20 was evaluated by separation of the n-hexane-in-water emulsion for 20 cyclic experiments. After each separation, the PAN/TiO_2_-PCL20 was washed with ethanol and dried at 40 °C for 3 h in an oven to remove the residual solvent.

### 2.5. Adsorption and Photocatalytic Experiments

To evaluate the adsorption process, PAN/TiO_2_-PCL20 was immersed in 50 mL RhB aqueous solution (12 mg L^−1^) and magnetically stirred under dark conditions for 60 min. 3.5 mL solution was extracted from the RhB solution at 15 min intervals to evaluate the adsorption capacity. The adsorption capacities were calculated by Equation (3).

(3) $$q=\frac{C_{i}-C_{t}}{m}\times V$$

where $C_{i}$ (mg L^−1^) and $C_{t}$ (mg L^−1^) were the initial concentration and the equilibrium concentration of RhB at time t. V (L) represented the volume of dye solution and m (mg) was the adsorbent dosage.

The photocatalytic activity of PAN/TiO_2_-PCL20 for RhB was measured through the following experiments. The PAN/TiO_2_-PCL20 was performed for RhB degradation under simulated solar irradiation for 80 min. A total of 3.5 mL liquid was extracted at 10 min intervals to evaluate removal rate. The removal rates (R%) of RhB were calculated by Equation (4).

(4) $$R(\%)=\frac{C_{0}-C_{t}}{C_{0}}\times 100$$

where $C_{0}$ (mg L^−1^) and $C_{t}$ (mg L^−1^) represented the initial and equilibrium concentration of the RhB organic dye, respectively.

## 3. Results and Discussion 

### 3.1. FT-IR Analysis

**Figure 1:**
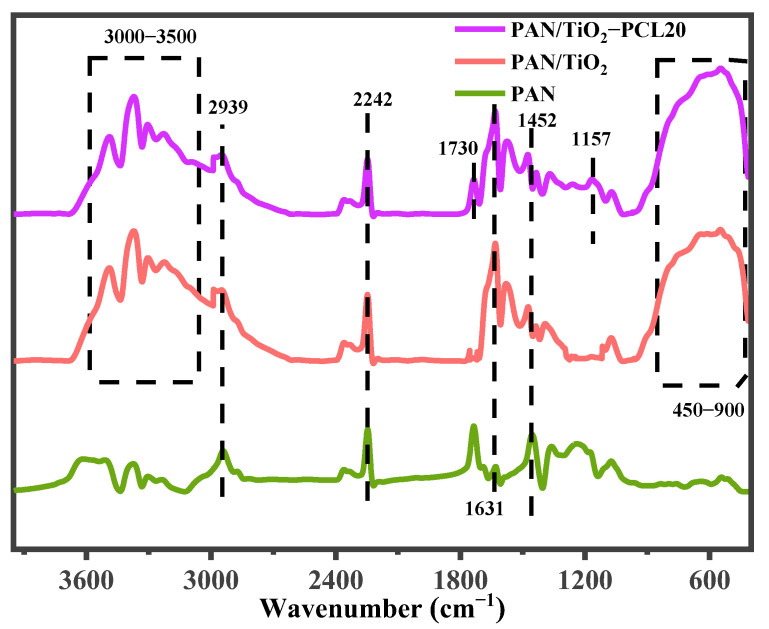
FTIR spectra of PAN, PAN/TiO_2_ and PAN/TiO_2_-PCL20. (Two dashed boxes represented the range of characteristic peaks).

The functional group characteristics of the PAN, PAN/TiO_2_, PAN/TiO_2_-PCL20 were investigated by FT-IR (Figure 1). The characteristic peaks of PAN were as follows: 1452 cm^−1^ and 2939 cm^−1^ (-CH_2_ symmetric and asymmetric stretching vibration), 2242 cm^−1^ (-C≡N stretching vibration) and 1631 cm^−1^ (-C=N stretching vibration) [39]. Compared with the FT-IR spectra of PAN, the broad band in the range of 3000–3500 cm^−1^ in PAN/TiO_2_ spectra corresponded to the stretching vibration of -OH, which was attributed to the introduction of TiO_2_ [40]. At the same time, the adsorption band at 450–900 cm^−1^ was attributed to the Ti-O bonds of TiO_2_ [41]. For PAN/TiO_2_-PCL20, the new peak that appeared at 1730 cm^−1^ belonged to the C=O stretching vibration, and the peak at 1157 cm^−1^ contributed to the C-O stretching vibration of PCL [42].

### 3.2. Surface Morphology Analysis 

**Figure 2:**
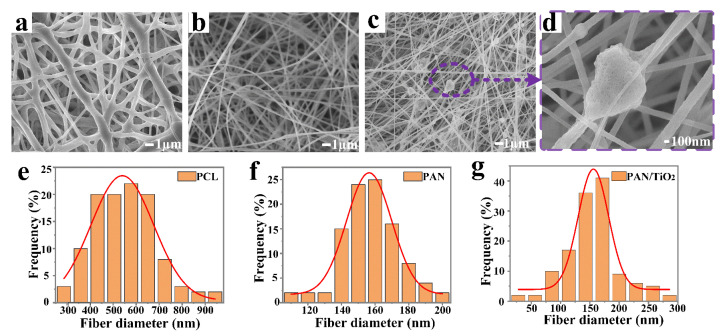
SEM of (**a**) PCL top layer, (**b**) PAN and (**c**,**d**) PAN/TiO_2_ substrate layer; Diameter distribution of (**e**) PCL top layer, (**f**) PAN and (**g**) PAN/TiO_2_ substrate layer.

As depicted in Figure 2, the surface morphologies of the PAN/TiO_2_-PCL20 were characterized by SEM images. The surface of PCL layer was relatively rough and the average fiber diameter was 553.7 ± 4.3 nm (Figure 2a,e). The PAN substrate layer had a smooth surface with an average fiber diameter of 158.4 ± 2.4 nm (Figure 2b,f). After the incorporation of TiO_2_ into PAN fibers, a large number of beaded structures appeared in PAN/TiO_2_ fibers and the PAN/TiO_2_ fiber diameter decreased to 153.8 ± 1.7 nm (Figure 2c,g). This was attributed to an increase in the viscosity and conductivity of the PAN/TiO_2_ precursor solution (Table 1). 

### 3.3. BET Analysis

**Figure 3:**
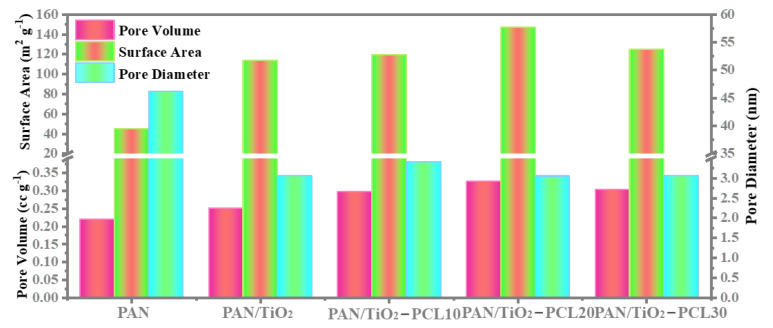
BET analysis of the PAN, PAN/TiO_2_ and PAN/TiO_2_-PCLx.

The Nitrogen (N_2_) adsorption-desorption isotherms for PAN, PAN/TiO_2_, PAN/TiO_2_-PCLx are shown in Figure S1, while the BET results are analyzed in Figure 3. The specific surface area and pore volume of pure PAN were 45.198 m^2^ g^−1^ and 0.220 cc g^−1^, respectively. Compared with pure PAN, the specific surface area and pore volume of PAN/TiO_2_ increased to 113.901 m^2^ g^−1^ and 0.251 cc g^−1^. This indicated that PAN/TiO_2_ had a smaller pore size and larger specific surface area, which further enhanced the hydrophilicity of PAN substrate [43]. After the incorporation of the PCL layer, the specific surface area and pore volume of PAN/TiO_2_-PCLx increased significantly. The PAN/TiO_2_-PCL20 had the largest specific surface area (147.377 m^2^ g^−1^) and pore volume (0.326 cc g^−1^). Compared with PAN, the PAN/TiO_2_-PCL20 Janus membrane appeared to have a large number of pore diameters smaller than 50 nm—this was confirmed by BET analysis, which showed the advantages for the separation of oil-water emulsions and the adsorption of RhB [44,45]. 

### 3.4. LSCM Analysis

**Figure 4:**
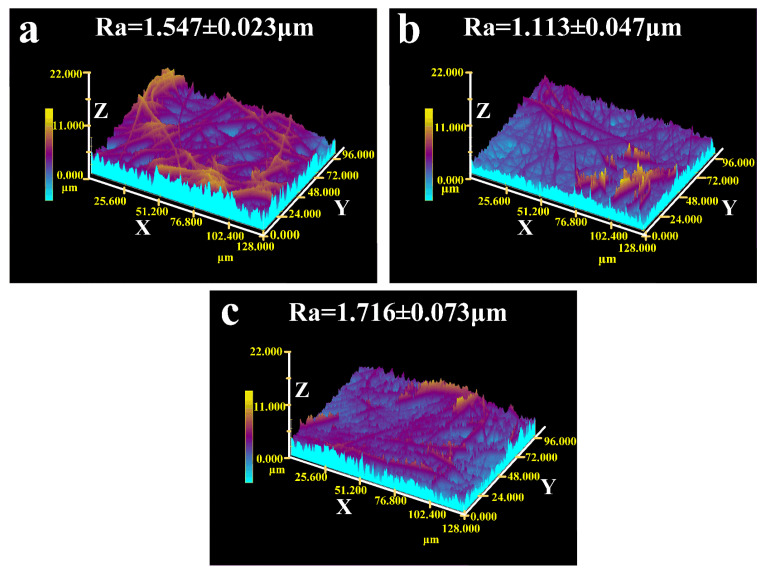
LSCM images of the (**a**) PCL top layer; (**b**) PAN layer; and (**c**) PAN/TiO_2_ substrate layer. (Ra: arithmetic mean height; X, Y, Z: the axis of 3D images).

The average surface roughness (Ra) of the PCL layer, and the PAN and PAN/TiO_2_ substrate layers is analyzed in Figure 4. The Ra of the PCL layer and PAN substrate layer was 1.547 ± 0.023 μm and 1.113 ± 0.047 μm, respectively (Figure 4a,b). Compared with the PAN substrate layer, the Ra of PAN/TiO_2_ substrate layer increased to 1.716 ± 0.073 μm (Figure 4c). It could be observed that the surface roughness of the PAN/TiO_2_ substrate layer improved significantly. Owing to the impact of PAN on the hydrophilic layer, the larger roughness for PAN/TiO_2_ could lead to a further improvement in hydrophilicity [46], which could further increase the asymmetric wettability of the Janus membrane when combined with BET analysis.

### 3.5. Surface Wettability

**Figure 5:**
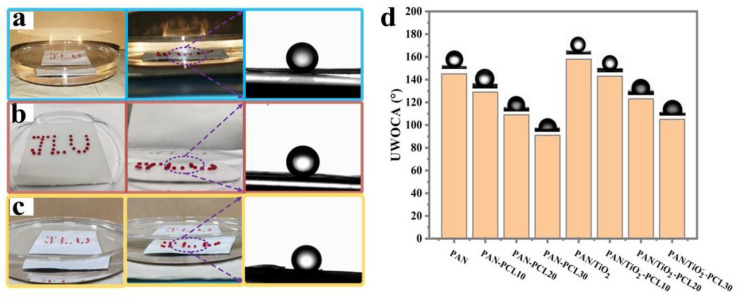
Oil droplets underwater onto (**a**) PAN, (**b**) PAN/TiO_2_, (**c**) PAN/TiO_2_-PCL20; (**d**) UOCAs onto PAN, PAN-PCLs and PAN/TiO_2_-PCLs with a different electrospinning time of PCL (10 min, 20 min, 30 min).

The surface wettability of Janus composite membranes was studied by measuring the underwater oil contact angle (UOCA). Figure S2 showed the dynamic wetting behaviors of water droplets on the PAN, PAN/TiO_2_ and PAN/TiO_2_-PCLx, and all prepared membranes exhibited hydrophilic layer in the air. As shown in Figure 5a–c, the oil droplets were all dispersed on the PAN, PAN/TiO_2_ and PAN/TiO_2_-PCL20 surface in a spherical shape, which indicated the three samples possessed underwater oleophobic properties. As displayed in Figure 5d, the UOCA of PAN was 144.6 ± 1.5°. The UOCAs of PAN-PCLx decreased from 129 ± 1.1° to 91 ± 1.2° as the PCL electrospinning time increased. After adding TiO_2_ to the PAN substrate layer, the UOCA of PAN/TiO_2_ increased to 158 ± 1.5°. The UOCAs of PAN/TiO_2_-PCLx decreased from 143 ± 1.3° to 105 ± 1.6° as the PCL electrospinning time increased. All the UOCAs of PAN/TiO_2_-PCLx were significantly higher than corresponding PAN-PCLx, which illustrated the hydrophilic layer modified by TiO_2_ enhanced the underwater oleophobic properties of the PAN/TiO_2_-PCLx Janus membrane. 

### 3.6. Oil/Water Separation

**Figure 6:**
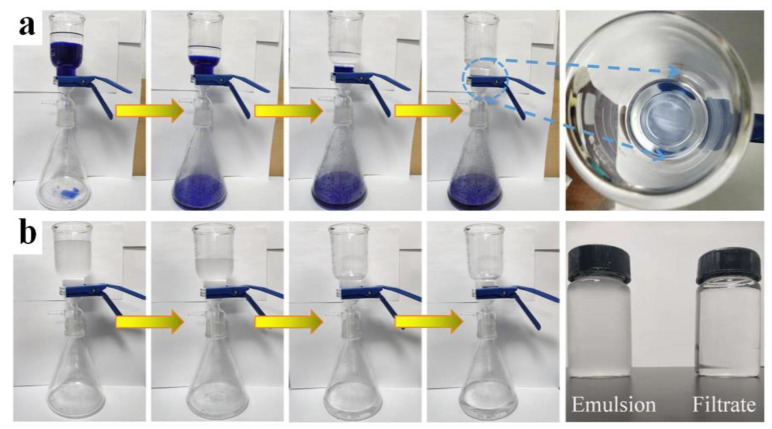
(**a**) Separation experiment of immiscible n-hexane-water mixture; (**b**) Separation experiment of n-hexane-in-water emulsion.

Figure 6a showed the separation procedure for the immiscible n-hexane-water mixture. During the separation process, water (methyl blue dyed) permeated the membrane and rapidly flowed into the collector below, while n-hexane was retained above the membrane surface. Figure 6b shows the separation procedure for surfactant-stabilized n-hexane-in-water emulsion by PAN/TiO_2_-PCL20. The emulsion showed the color of milky white before purification and became clear and transparent after filtration.

**Figure 7:**
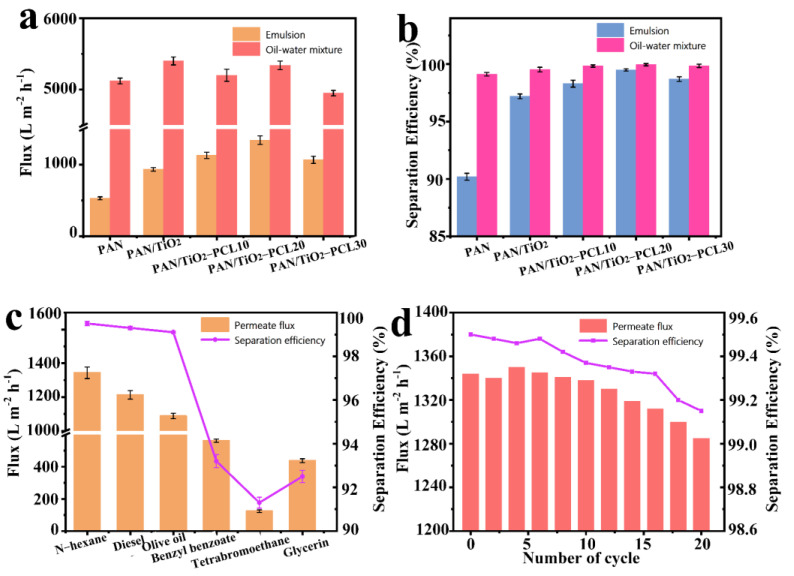
(**a**) Permeate fluxes and (**b**) separation efficiencies of different membranes for immiscible n-hexane-water mixture and n-hexane-in-water emulsion; (**c**) Permeate fluxes and separation efficiencies of PAN/TiO_2_-PCL20 for different types of oil-in-water emulsions; (**d**) Permeate fluxes and separation efficiencies of PAN/TiO_2_-PCL20 for n-hexane-in-water emulsion in 20 cycles.

Figure 7a,b shows the separation performance of n-hexane-water mixture and n-hexane-in-water emulsion by different membranes. The permeate fluxes and separation efficiencies of PAN/TiO_2_ for mixture and emulsion were all higher than PAN. For PAN/TiO_2_-PCLx, the permeate fluxes and separation efficiencies for mixture and emulsion all increased rapidly and then decreased with the increase of PCL layer content. In summary, the PAN/TiO_2_-PCL20 possessed the highest permeate fluxes (5340 ± 60 L m^−2^ h^−1^ for n-hexane-water mixture, 1344 ± 35 L m^−2^ h^−1^ for n-hexane-in-water emulsion) and separation efficiencies (99.95% for n-hexane-water mixture, 99.52% for n-hexane-in-water emulsion). 

Thus, PAN/TiO_2_-PCL20 was selected to carry out the following separation experiments for different types of oil-in-water emulsions. N-hexane, diesel oil, olive oil, benzyl benzoate, 1,1,2,2-tetrabromoethane and glycerol were chosen to prepare the oil-in-water emulsions, and the separation efficiencies for these oil-in-water emulsions by PAN/TiO_2_-PCL20 were all higher than 92.5%. Especially in the case of n-hexane-in-water emulsion, the permeate flux and efficiency reached 1344 ± 35 L m^−2^ h^−1^ and 99.52%, respectively (Figure 7c). The different separation performance for different oil-in-water emulsions might be ascribed to the viscosity and density of the oils [47]. More importantly, the durability test demonstrated that there was no significant decrease in permeate flux, and the separation efficiency of hexane-in-water emulsion for PAN/TiO_2_-PCL20 still remained above 99.15%, even after 20 cycles of separation (Figure 7d). The as-prepared PAN/TiO_2_-PCL20 had superior reusability of oil-in-water emulsions than other reported separation materials (see Table 2).

**Figure 8:**
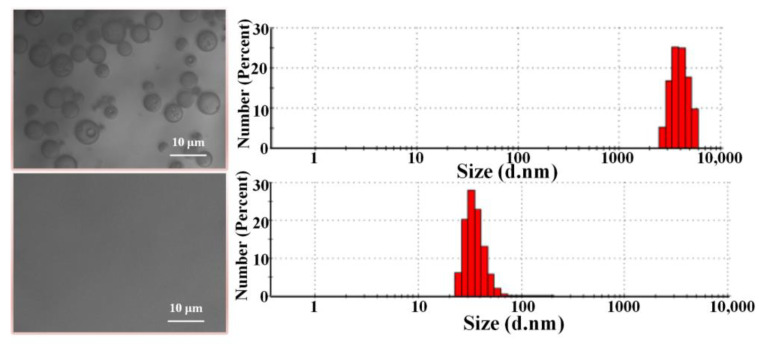
Optical microscope images and DLS analysis, both before and after separation of n-hexane-in-water emulsion.

Figure 8 shows the optical microscope images and DLS analysis of PAN/TiO_2_-PCL20m both before and after separation for n-hexane-in-water emulsion. The optical microscope images revealed that, prior to separation, the oil droplets were evenly spread in the emulsion with an average particle size of 4.03 ± 0.05 μm. After filtration, there were no apparent oil droplets in the filtrate, and few droplets were examined with an average particle size of 138.8 ± 5 nm. 

### 3.7. Oil/Water Separation Mechanism

The mechanism of oil/water separation was summarized in accordance with the preceding discussion. Compared to PAN and PAN-PCLx membranes, the PAN/TiO_2_-PCL20 exhibited excellent oil/water separation performance, which could be ascribed to its asymmetric wettability and hierarchical structure. This asymmetric wettability could be explained by the Young-Laplace Equation (5) [53]:

(5) $$\delta P=\frac{2\gamma \cos\theta }{R}$$

where δP was the capillary pressure, γ was the water surface tension, θ was the water contact angle and R was the pore radius.

**Figure 9:**
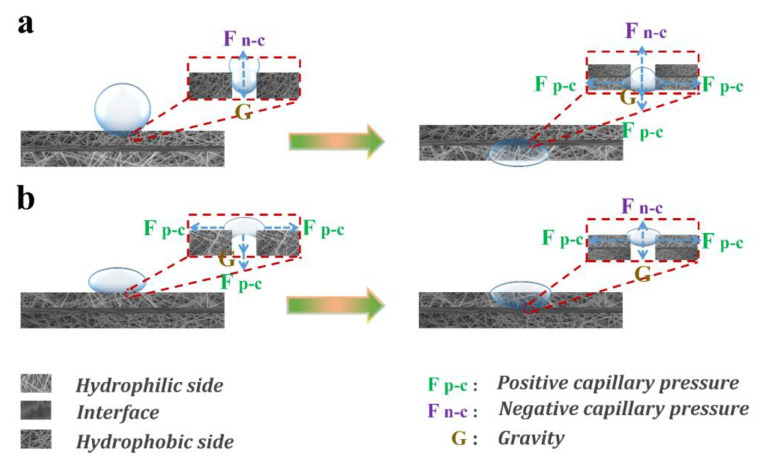
Schematic diagram of the oil/water separation and unidirectional permeation mechanism of PAN/TiO_2_-PCL20. ((**a**): the PCL faced to water and separated emulsion, (**b**): the PAN/TiO_2_ faced to water and resisted water).

When the PCL layer faced up to and operated in oil/water separation experiments, the hydrophobic layer provided negative capillary pressure (F_n-c_) and the hydrophilic layer provided positive capillary pressure (F_p-c_). When δP within the composite membrane was positive, water could penetrate successfully [54]. When water droplets were in contact with the surface of the hydrophobic PCL layer, it would be subjected to two opposing forces (the water gravity G and F_n-c_). As the quantity of the water droplet increased, the value of G increased accordingly. When G > F_n-c_, the water droplets could penetrate through the hydrophobic PCL layer to the PAN-based substrate layer, and the water then penetrated through the PAN/TiO_2_-PCL20 by F_p-c_. PAN/TiO_2_ substrate had better hydrophilicity than PAN substrate due to its larger specific surface area and roughness, which could enhance asymmetric wettability for the Janus membrane and generate the larger F_p-c_. Meanwhile, compared with the PAN substrate, the hydration layer formed in the PAN/TiO_2_ substrate improved the oleophobicity of PAN/TiO_2_-PCL20 and resisted oil penetration (Figure 9a)—all of these developments contributed to efficient emulsion separation performance. 

### 3.8. Removal of RhB Organic Dye

**Figure 10:**
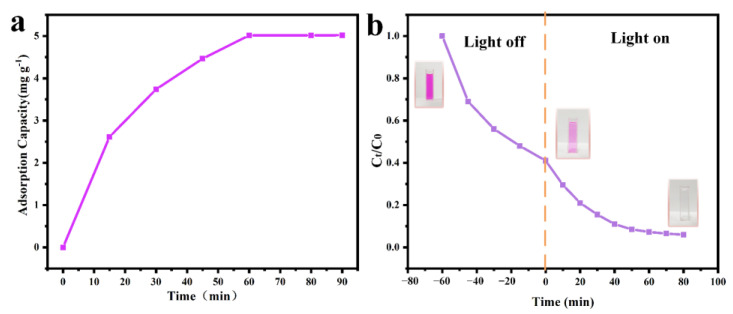
(**a**) Effect of adsorption time on adsorption capacity of PAN/TiO_2_-PCL20; (**b**) Removal performance of PAN/TiO_2_-PCL20 of RhB under simulated solar irradiation.

RhB was chosen as the representative stain for evaluating the removal performance of the PAN/TiO_2_-PCL20. As displayed in Figure 10a, PAN/TiO_2_-PCL20 exhibited adsorption capacity with 5.02 mg g^−1^ for RhB within 60 min of adsorption equilibria under a dark condition (see Figure 10a). The removal rate of RhB by PAN/TiO_2_-PCL20 achieved 59%t adsorption equilibria. When the removal experiment for RhB by PAN/TiO_2_-PCL20 was exposed to the simulated solar irradiation, the removal rate of PAN/TiO_2_-PCL20 for RhB could reach 93.2% (Figure 10b). The reason for this could be because, under simulated solar irradiation, TiO_2_ generated hydroxyl radicals and superoxide radicals to degrade RhB dye molecules into carbon dioxide and water on the PAN/TiO_2_-PCL20 [55,56]. This could provide evidence that PAN/TiO_2_-PCL20 exhibited photocatalytic performance for RhB under the simulated solar irradiation. The inset of the Figure 10b shows the color change of the RhB solution at different time intervals, and it should be noticed that the color of the RhB solution became transparent after adsorption and photocatalytic degradation.

### 3.9. Unidirectional Water Transfer

**Figure 11:**
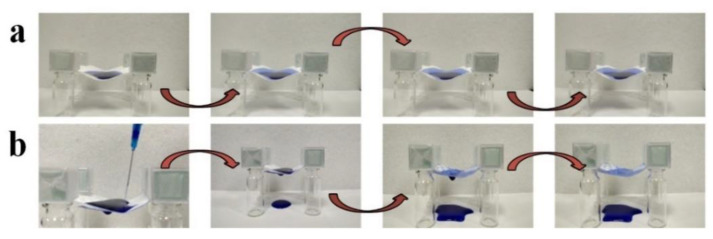
Unidirectional water transfer process of PAN/TiO_2_-PCL20: (**a**) PAN/TiO_2_ side facing water and (**b**) PCL side facing water.

The asymmetric wettability of membranes might result in unidirectional water transfer capability [53]. The unidirectional water transfer capability of PAN/TiO_2_-PCL20 was further studied. When the hydrophilic PAN/TiO_2_ layer faced up, water merely spread on the surface of PAN/TiO_2_-PCL20 (Figure 11a). When the hydrophobic PCL layer faced up, water could spontaneously pass through PAN/TiO_2_-PCL20 (Figure 11b). PAN/TiO_2_-PCL20 exhibited unidirectional water transfer capability. The possible mechanism of unidirectional water transfer could also be explained, as illustrated in Figure 9: when the hydrophilic PAN/TiO_2_ layer faced up, water droplets continuously diffused to the surface of the membrane and formed a thin water layer. G value per unit area of water was less than the upward force to the water droplets provided by the hydrophobic PCL layer, meaning water would be blocked on the surface of PAN/TiO_2_-PCL20 (Figure 9b). When the hydrophobic PCL layer faced up, G value per unit area of water was higher than the repulsive force provided by the hydrophobic side counterpart. When G > F_n-c_, water droplets passed through the PAN/TiO_2_-PCL20 by gravity and the capillary force of the substrate (Figure 9a). The result showed that the asymmetric wettability of PAN/TiO_2_-PCL20 exhibited unidirectional water transfer capability within a certain water pressure range [57,58]. 

### 3.10. Mechanical Properties

**Figure 12:**
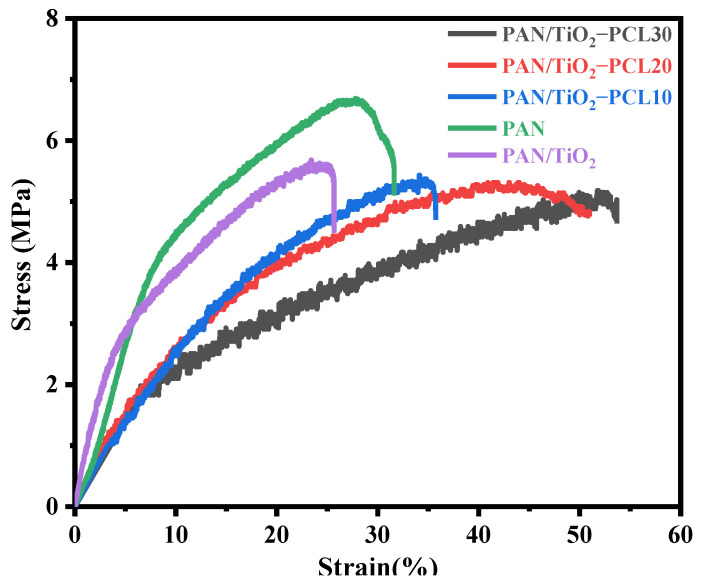
Stress-Strain curves of PAN, PAN/TiO_2_ and PAN/TiO_2_-PCLx.

The mechanical performance of the separator was also necessary in the practical application for oil/water separation. Figure 12 shows the stress-strain curves of PAN, PAN/TiO_2_ and PAN/TiO_2_-PCLx. The tensile strength and train of PAN was 6.69 MPa and 28.67%, respectively. Compared with pure PAN, the tensile strength and strain of PAN/TiO_2_ substrate layer were reduced to 5.68 MPa and 25.33%, respectively, which might be attributed to the existence of bead structures and porosities in the PAN/TiO_2_. Compared to PAN/TiO_2_, the PAN/TiO_2_-PCLx possessed superior mechanical properties. With the increase of PCL content, PAN/TiO_2_-PCLx exhibited a decrease in tensile strength and an increase in strain. The PAN/TiO_2_-PCL30 had the most outstanding mechanical property and the maximum strain reached 53.67%. The result indicated that PAN/TiO_2_-PCLx all had good mechanical performance for oil/water separation (Figure S3).

## 4. Conclusions

In summary, an environmentally friendly PAN-based Janus composite membrane with asymmetric wettability and hierarchical structure was successfully fabricated. The addition of TiO_2_ could further enhance the hydrophilicity of the hydrophilic PAN substrate by increasing the specific surface area and roughness, which further improved the asymmetric wettability of the Janus membrane. The prepared PAN/TiO_2_-PCL20 exhibited excellent oil/water separation performance for diverse surfactant-stabilized oil-in-water emulsions. Even after 20 cycles, the separation efficiency for n-hexane-in-water emulsion remained above 99.15%, which exhibited the excellent reusability for an environmentally friendly separator. Meanwhile, the PAN/TiO_2_-PCL20 also had superior adsorption (5.02 mg g^−1^) and removal performance (93.2%) for RhB. In addition, the PAN/TiO_2_-PCL20 had outstanding unidirectional water transfer capability and mechanical property. However, the corrosion resistance of PCL layer was expected to be further improved. To address this problem, the corrosion-resistant inorganic and organic component may be used to functionalize PCL modification by subsequent studies.

## Figures and Tables

**Table 1:** The physical parameters of electrospinning solutions.

Sample	Viscosity (mPa s)	Conductivity (µS cm^−1^)
PCL	360.2	0.351
PAN	85.5	34.2
PAN/TiO_2_	125.4	67.8

**Table 2:** Reusability of the other reported oil-in-water emulsion separation materials.

Material	Flux (L m^−2^ h^−1^)	Efficiency (%)	Reference
PAN/TiO_2_-PCL20	1285	99.15	This work
Waste PET plastics	1197	95	[48]
PVDF/TiO_2_	1398	99	[49]
PVP-VTES	17.45	91.47	[50]
PDMS@SiO_2_@UiO66-OSiR	970	98.64	[51]
F-PPS@TiO_2_	950	98.4	[52]

## Data Availability

Data presented in this article are available at request from the corresponding author.

## Supplementary Materials

The following supporting information can be downloaded at: https://www.mdpi.com/article/10.3390/ma17020417/s1, Figure S1: Nitrogen (N2) adsorption-desorption isotherms of (a) PAN, PAN/TiO2, (b) PAN/TiO2-PCL10, (c) PAN/TiO2-PCL20 and (d) PAN/TiO2-PCL30. Figure S2: Dynamic optical images of the wettability of a water droplet on (a) PAN, (b) PAN/TiO_2_, (c) PAN/TiO_2_-PCL10, (d) PAN/TiO_2_-PCL20 and (e) PAN/TiO_2_-PCL30. Figure S3: Stress-strain curve of PCL. References [59,60] are cited in the supplementary materials.

## References

[1] Berako Belachew G., Hu C.C., Wang C.F., Liao M.Y., Hung W.S., Chen J.K., Lai J.Y. (2024). Preparation of citric acid-modified cellulose materials with superwetting and antibacterial characteristics for highly-efficient separation of oil/water mixtures, emulsions, and dye-containing wastewater. Sep. Purif. Technol..

[2] Sutar R.S., Latthe S.S., Jundle A.R., Gaikwad P.P., Ingole S.S., Nagappan S., Kim Y.H., Bhosale A.K., Saji V.S., Liu S. (2024). A facile approach for oil-water separation using superhydrophobic polystyrene-silica coated stainless steel mesh bucket. Mar. Pollut. Bull..

[3] Sahu P.S., Verma R.P., Tewari C., Sahoo N.G., Saha B. (2023). Facile fabrication and application of highly efficient reduced graphene oxide (rGO)-wrapped 3D foam for the removal of organic and inorganic water pollutants. Environ. Sci. Pollut. Res..

[4] Hasiri M., Kantzas A. (2024). Efficiency of oil separation and demulsification following sonication gel degradation: Influence of Cr (III) ions, NaCl concentrations, and sodium-based retarders. Fuel.

[5] Saini H., Otyepková E., Schneemann A., Zbořil R., Otyepka M., Fischer R.A., Jayaramulu K. (2022). Hierarchical porous metal–organic framework materials for efficient oil–water separation. J. Mater. Chem..

[6] Hamedi H., Rezaei N., Zendehboudi S. (2022). A comprehensive review on demulsification using functionalized magnetic nanoparticles. J. Clean. Prod..

[7] Iskandar M.A., Yahya E.B., Abdul Khalil H.P.S., Rahman A.A., Ismail M.A. (2022). Recent Progress in Modification Strategies of Nanocellulose-Based Aerogels for Oil Absorption Application. Polymers.

[8] Oh S., Bang J., Jin H.J., Kwak H.W. (2023). Green Fabrication of Underwater Superoleophobic Biopolymeric Nanofibrous Membranes for Effective Oil–Water Separation. Adv. Fiber Mater..

[9] Gkinali A.A., Matsakidou A., Paraskevopoulou A. (2023). Assessing the emulsifying properties of Tenebrio molitor larvae protein preparations: Impact of storage, thermal, and freeze-thaw treatments on o/w emulsion stability. Int. J. Biol. Macromol..

[10] Scharnberg A.R.A., Oliveira H.A., Weschenfelder S.E., Rubio J., Azevedo A.C. (2023). Flocculation of emulsified oil-in-water with dodecylbenzene sulfonate and polyacrylamide and floc separation by dissolved air flotation. Colloids Surf. A Physicochem. Eng. Asp..

[11] Abu-Thabit N.Y., Kalam Azad A., Mezghani K., Akhtar S., Saeed Hakeem A., Drmosh Q.A., Yusuf Adesina A. (2024). Rapid, Sustainable, and Versatile Strategy Towards Fabricating Superhydrophobic Cotton Textile Membranes for Separation of Emulsified and Stratified Oil/Water Mixtures. Sep. Purif. Technol..

[12] Ortiz-Oliveros H.B., Flores-Espinosa R.M. (2020). Design of a mobile dissolved air flotation system with high rate for the treatment of liquid radioactive waste. Process Saf. Environ. Prot..

[13] Ihsanullah I., Bilal M., Sajid M., Mohammad A.W., Atieh M.A., Ghaffour N. (2024). Emerging MXenes: Revolutionizing oily wastewater treatment-a comprehensive and critical review. Sep. Purif. Technol..

[14] Mittag A., Rahman M.M., Hafez I., Tajvidi M. (2022). Development of Lignin-Containing Cellulose Nanofibrils Coated Paper-Based Filters for Effective Oil-Water Separation. Membranes.

[15] Myeong J., Deshmukh P.R., Gyu Shin W. (2023). Facile preparation of superhydrophilic and underwater superoleophobic stainless steel mesh for oil–water separation. J. Ind. Eng. Chem..

[16] Diyana Suzaimi N., Sean Goh P., Chun Wong K., Taniguchi T., Wei Lim J., Ahmad Nizam Nik Malek N., Fauzi Ismail A. (2024). Construction of 1D akageneite-templated nanochannels in polyamide forward osmosis membrane for high flux separation and nutrient enrichment. Sep. Purif. Technol..

[17] Mallya D.S., Abdikheibari S., Dumée L.F., Muthukumaran S., Lei W., Baskaran K. (2023). Removal of natural organic matter from surface water sources by nanofiltration and surface engineering membranes for fouling mitigation—A review. Chemosphere.

[18] Imsong R., Dhar Purkayastha D. (2023). Dual-functional superhydrophilic/underwater superoleophobic 2D Ti_3_C_2_T_X_ MXene-PAN membrane for efficient oil-water separation and adsorption of organic dyes in wastewater. Sep. Purif. Technol..

[19] Han L., Shen L., Lin H., Huang Z., Xu Y., Li R., Li B., Chen C., Yu W., Teng J. (2023). 3D printing titanium dioxide-acrylonitrile-butadiene-styrene (TiO_2_-ABS) composite membrane for efficient oil/water separation. Chemosphere.

[20] Polak D., Zielińska I., Szwast M., Kogut I., Małolepszy A. (2021). Modification of Ceramic Membranes with Carbon Compounds for Pharmaceutical Substances Removal from Water in a Filtration-Adsorption System. Membranes.

[21] Zielińska I., Polak D., Szwast M. (2021). Analysis of the adsorption of selected pharmaceuticals on a composite material PEBAX/GO. J. Water Process Eng..

[22] Annam Renita A., Sathish S., Kumar P.S., Prabu D., Manikandan N., Mohamed Iqbal A., Rajesh G., Rangasamy G. (2023). Emerging aspects of metal ions-doped zinc oxide photocatalysts in degradation of organic dyes and pharmaceutical pollutants-A review. J. Environ. Manag..

[23] Parasuraman B., Kandasamy B., Murugan I., Alsalhi M.S., Asemi N., Thangavelu P., Perumal S. (2023). Designing the heterostructured FeWO_4_/FeS_2_ nanocomposites for an enhanced photocatalytic organic dye degradation. Chemosphere.

[24] Zhao Y., Fan X., Zheng H., Liu E., Fan J., Wang X. (2024). Bi_2_WO_6_/AgInS_2_ S-scheme heterojunction: Efficient photodegradation of organic pollutant and toxicity evaluation. J. Mater. Sci. Technol..

[25] Rego R.M., Kurkuri M.D., Kigga M. (2022). A comprehensive review on water remediation using UiO-66 MOFs and their derivatives. Chemosphere.

[26] Gupta S., Gomaa H., Ray M.B. (2021). Performance characterization of a hybrid adsorptive-photocatalytic (APC) oscillatory membrane reactor for micropollutant removal. Sep. Purif. Technol..

[27] Baig U., Matin A., Gondal M.A., Zubair S.M. (2019). Facile fabrication of superhydrophobic, superoleophilic photocatalytic membrane for efficient oil-water separation and removal of hazardous organic pollutants. J. Clean. Prod..

[28] Wu H., Shi J., Ning X., Long Y., Zheng J. (2022). The High Flux of Superhydrophilic-Superhydrophobic Janus Membrane of cPVA-PVDF/PMMA/GO by Layer-by-Layer Electrospinning for High Efficiency Oil-Water Separation. Polymers.

[29] Afsari M., Shirazi M.M.A., Ghorbani A.H., Sayar O., Shon H.K., Tijing L.D. (2023). Triple-layer nanofiber membrane with improved energy efficiency for treatment of hypersaline solution via membrane distillation. J. Environ. Chem. Eng..

[30] Selvam A., Raj Singh Y., Karna A., Bhatnagar A., Mukherjee M., Chakrabarti S. (2023). Emergence of the Janus-MOF (J-MOF) Boat as a Nascent Amalgamation in the Arena of Photothermal Desalination. ACS Appl. Mater. Interfaces.

[31] Li X., Ma Q., Tian J., Xi X., Li D., Dong X., Yu W., Wang X., Wang J. (2017). Double anisotropic electrically conductive flexible Janus-typed membranes. Nanoscale.

[32] Li H., Yang J., Xu Z. (2020). Asymmetric Surface Engineering for Janus Membranes. Adv. Mater. Interfaces.

[33] Agaba A., Marriam I., Tebyetekerwa M., Yuanhao W. (2021). Janus hybrid sustainable all-cellulose nanofiber sponge for oil-water separation. Int. J. Biol. Macromol..

[34] Nambikkattu J., Kaleekkal N.J. (2023). Development of Janus membranes with asymmetric wettability for desalination of impaired seawater by direct contact membrane distillation. J. Environ. Chem. Eng..

[35] Abuhantash F., Abuhasheesh Y.H., Hegab H.M., Aljundi I.H., Al Marzooqi F., Hasan S.W. (2023). Hydrophilic, oleophilic and switchable Janus mixed matrix membranes for oily wastewater treatment: A review. J. Water Process Eng..

[36] Naseem S., Wu C.-M., Motora K.G. (2021). Novel multifunctional Rb_x_WO_3_@Fe_3_O_4_ immobilized Janus membranes for desalination and synergic-photocatalytic water purification. Desalination.

[37] Chen K., Zhu J., Tan Y., Sun F., Gan J., Peng H., Zhan T., Lyu J. (2022). Development of gradient-wetting Janus wood membrane with high-efficiency fog collection and oil-water separation. Chem. Eng. J..

[38] Pan T.D., Li Z.J., Shou D.H., Shou W., Fan J.T., Liu X., Liu Y. (2019). Buoyancy Assisted Janus Membrane Preparation by ZnO Interfacial Deposition for Water Pollution Treatment and Self-cleaning. Adv. Mater. Interfaces.

[39] Cintra I.L.R., Rezende M.C., Guerrini L.M., Nahra L.R., Lucas R.R., Montagna L.S., Botelho E.C. (2023). Electrospinning of PAN/lignin blends aiming the production of carbon nanofibers. MRS Commun..

[40] Taghizadeh Lendeh P., Sarrafi A.H.M., Alihosseini A., Bahri-Laleh N. (2024). Synthesis and characterization ZnO/TiO_2_ incorporated activated carbon as photocatalyst for gas refinery effluent treatment. Polyhedron.

[41] Paul R., Kavinarmatha K., Parthiban S. (2023). Tantalum doped titanium dioxide nanoparticles for efficient photocatalytic degradation of dyes. J. Mol. Struct..

[42] He N., Li L., Chen J., Zhang J., Liang C. (2021). Extraordinary Superhydrophobic Polycaprolactone-Based Composite Membrane with an Alternated Micro–Nano Hierarchical Structure as an Eco-friendly Oil/Water Separator. ACS Appl. Mater. Interfaces.

[43] Wang X., Li B., Zhou L., Ma J., Zhang X., Li H., Liang C., Liu S., Wang H. (2018). Influence of surface structures on biocompatibility of TiO_2_ /HA coatings prepared by MAO. Mater. Chem. Phys..

[44] Kong L., Li Y., Qiu F., Zhang T., Guo Q., Zhang X., Yang D., Xu J., Xue M. (2018). Fabrication of hydrophobic and oleophilic polyurethane foam sponge modified with hydrophobic Al_2_O_3_ for oil/water separation. J. Ind. Eng. Chem..

[45] Liu Z., Liu L., Liu J., Zhang W., Zhou T., Ji X. (2016). Enhanced photocatalytic performance of Er-doped Bi_24_O_31_Br_10_: Facile synthesis and photocatalytic mechanism. Mater. Res. Bull..

[46] Hovish M.Q., Hilt F., Rolston N., Xiao Q., Dauskardt R.H. (2019). Open Air Plasma Deposition of Superhydrophilic Titania Coatings. Adv. Funct. Mater..

[47] Parsaie A., Mohammadi-Khanaposhtani M., Riazi M., Tamsilian Y. (2020). Magnesium stearate-coated superhydrophobic sponge for oil/water separation: Synthesis, properties, application. Sep. Purif. Technol..

[48] Chen H., Zuo Z., Tian Q., Xue S., Qiu F., Peng X., Zhang T. (2023). Waste to treasure: A superwetting fiber membrane from waste PET plastic for water-in-oil emulsion separation. J. Clean. Prod..

[49] Sun F., Li T.-T., Ren H.-T., Shiu B.-C., Peng H.-K., Lin J.-H., Lou C.-W. (2021). Dopamine-decorated lotus leaf-like PVDF/TiO_2_ membrane with underwater superoleophobic for highly efficient oil-water separation. Process Saf. Environ. Prot..

[50] Zhang R., Deng C., Hou X., Li T., Lu Y., Liu F. (2023). Preparation and Characterization of a Janus Membrane with an “Integrated” Structure and Adjustable Hydrophilic Layer Thickness. Membranes.

[51] Zhai G., Wu J., Yuan Z., Li H., Sun D. (2023). Robust Superhydrophobic PDMS@SiO_2_@UiO66-OSiR Sponge for Efficient Water-in-Oil Emulsion Separation. Inorg. Chem..

[52] Yang C., Han N., Han C., Wang M., Zhang W., Wang W., Zhang Z., Li W., Zhang X. (2019). Design of a Janus F-TiO_2_@PPS Porous Membrane with Asymmetric Wettability for Switchable Oil/Water Separation. ACS Appl. Mater. Interfaces.

[53] Yalishev S.V., Abbasi N.A., Iqbal M., Alnaser A.S. (2023). Comparing Water Transport Properties of Janus Membranes Fabricated from Copper Mesh and Foam Using a Femtosecond Laser. Langmuir.

[54] Balachandran A., Bhat I.M., Tabasum R., Mohd G., Wani M.F., Majid K., Wahid M., Lone S. (2023). Transfer-Printed Environmental-Friendly Anisotropic Filter with Laser-Controlled Micropores for Efficient Oil/Water Separation. ACS Appl. Polym. Mater..

[55] Safajou H., Khojasteh H., Salavati-Niasari M., Mortazavi-Derazkola S. (2017). Enhanced photocatalytic degradation of dyes over graphene/Pd/TiO_2_ nanocomposites: TiO_2_ nanowires versus TiO_2_ nanoparticles. J. Colloid Interface Sci..

[56] Rozenman G.G., Peisakhov A., Zadok N. (2022). Dispersion of organic exciton polaritons—A novel undergraduate experiment. Eur. J. Phys..

[57] Chakraborty T., Kanth P.C., Trivedi M.U., Tripathi B., Pandey M.K. (2021). Fabrication of Janus type bi-layer polymeric membranes for advance water purification. Mater. Today Proc..

[58] Hosseini Z., Kargari A. (2024). Fabrication and characterization of hydrophobic/hydrophilic dual-layer polyphenyl sulfone Janus membrane for application in direct contact membrane distillation. Desalination.

[59] Kılıç D., Sevim M., Eroğlu Z., Metin Ö., Karaca S. (2021). Strontium oxide modified mesoporous graphitic carbon nitride/titanium dioxide nanocomposites (SrO-mpg-CN/TiO_2_) as efficient heterojunction photocatalysts for the degradation of tetracycline in water. Adv. Powder Technol..

[60] Liu G., Wang G., Hu Z., Su Y., Zhao L. (2019). Ag_2_O nanoparticles decorated TiO_2_ nanofibers as a p-n heterojunction for enhanced photocatalytic decomposition of RhB under visible light irradiation. Appl. Surf. Sci..

