# Supplementary material for: The Design of PAN-Based Janus Membrane with Adjustable Asymmetric Wettability in Wastewater Purification
Source: Materials (Basel). 2024 Jan 14;17(2):417. doi: 10.3390/ma17020417 (PMC10817498; doi:10.3390/ma17020417)
Supplement: Supplementary file 1 [file materials-17-00417-s001.zip › materials-2804050-supplementary.pdf]

Supporting Information

The Design of PAN-Based Janus Membrane with Adjustable Asymmetric Wettability in Wastewater Purification

Yuehui Wang [1,†], Jun Huang [1,†], Ye Zhang [1], Shiwen Zhang [1], Lili Li [1,*] and Xuan Pang [2,*]

[1] Key Laboratory of Automobile Materials, Ministry of Education, College of Materials Science and Engineering, Jilin University, Changchun 130022, China; 15161110960@163.com (Y.W.); 17843334500@163.com (J.H.); 15124698738@163.com (Y.Z.); shiwenuknow@163.com (S.Z.)

[2] Key Laboratory of Polymer Ecomaterials, Changchun Institute of Applied Chemistry, Chinese Academy of Sciences, Changchun 130022, China

* Correspondence: lilyllee@jlu.edu.cn (L.L.); xpang@ciac.ac.cn (X.P.)

† These authors contributed equally to this work.

1. Preparation of PAN−PCL Janus membrane

1 g PAN was dissolved in 10 mL DMF by magnetic stirring for 12 h at room temperature, and then PAN precursor solution was obtained. At the same time, 2 g PCL was added into 10 mL CF/DMF (4:1, v/v) mixed solvent to obtain 20 wt% PCL precursor solution. Both precursor solutions were placed into 5 mL syringes. The PAN substrate layer was fabricated at 16 kV applied voltage and 1 mL h^{-1} feeding speed for 2 h. Then the PCL fiber membranes with different thickness were electrospun on PAN substrate layer with an applied voltage of 12 kV and a flow rate of 2 mL h^{-1} for different electrospinning time (10 min, 20 min and 30 min, respectively). The prepared Janus membranes were named PAN−PCLx, where x represented the electrospinning time of PCL.

2. BET analysis

As presented in Figure S1a, pure PAN exhibited type III adsorption-desorption isotherm with the H_1 hysteresis loop, implying the existence of mesoporous structure [1]. PAN/TiO_2 and PAN/TiO_2−PCLx all possessed typical type II isotherms with sharp H_3 hysteresis loop and the adsorption capacities were all higher than pure PAN (Figure S1a−d) [2]. Meanwhile, the adsorption capacities of PAN/TiO_2−PCLx increased with the introduction of PCL, and the PAN/TiO_2−PCL20 exhibited the maximum adsorption capacity.

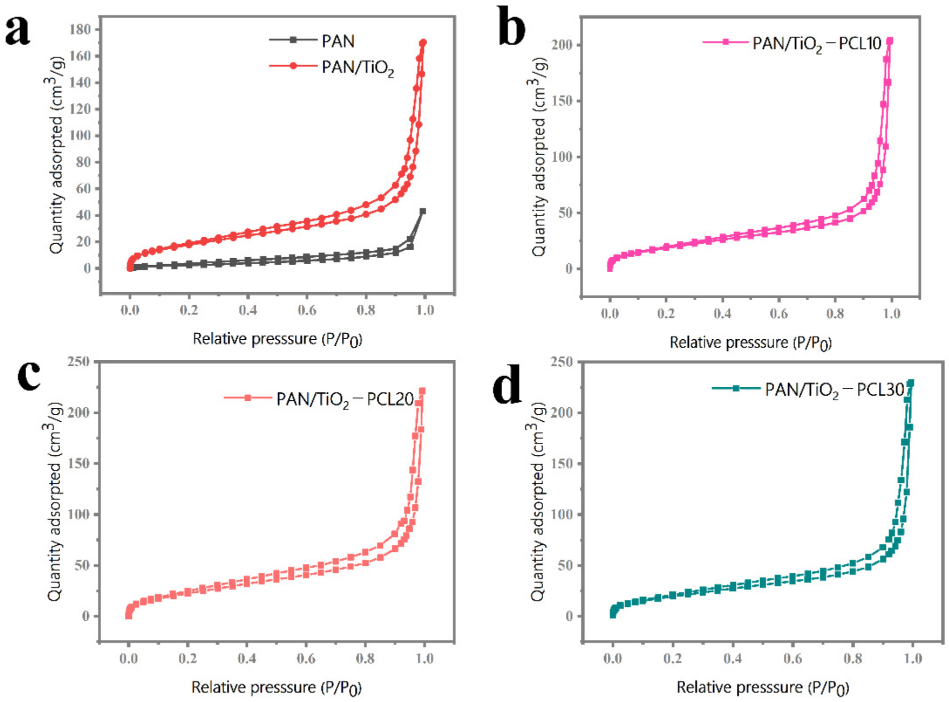

Figure S1. Nitrogen (N_2) adsorption-desorption isotherms of (a) PAN, PAN/TiO_2, (b) PAN/TiO_2−PCL10, (c) PAN/TiO_2−PCL20 and (d) PAN/TiO_2−PCL30.

3. Surface wettability

Figure S2 was the dynamic wettability of a water droplet on different membranes. When the water droplet was in contact with the surface of PAN, the WCA was 94.2°. It took only 1.2 s to completely wet the surface of the PAN. While the water droplet immediately wetted the PAN/TiO_2, and the WCA of PAN/TiO_2 was 0°. The WCAs and wetting time of all PAN/TiO_2−PCL samples increased with the addition of PCL layer. The PAN/TiO_2−PCL30 possessed the highest WCA with 132.3° and the complete wetting time increased to 118 s. The result indicated that the introduction of TiO_2 and PCL could increase asymmetric wettability.

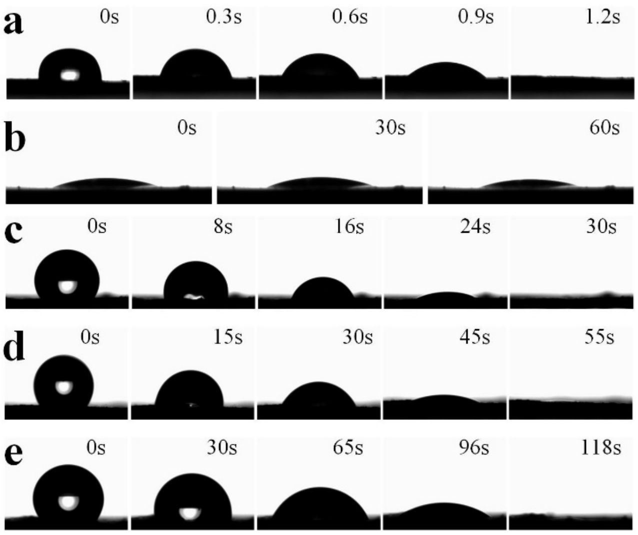

Figure S2. Dynamic optical images of the wettability of a water droplet on (a) PAN, (b) PAN/TiO_2, (c) PAN/TiO_2–PCL10, (d) PAN/TiO_2–PCL20 and (e) PAN/TiO_2–PCL30.

4. Mechanical properties

Figure S3 showed the stress–strain curve of PCL. The tensile strength and strain of PCL were 4.98 MPa and 99.23%, respectively.

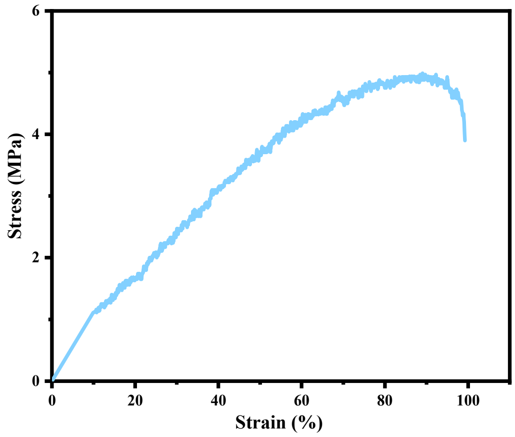

Figure S3. Stress–strain curve of PCL.

References

1. Kılıç, D.; Sevim, M.; Eroğlu, Z.; Metin, Ö.; Karaca, S., Strontium oxide modified mesoporous graphitic carbon nitride/titanium dioxide nanocomposites (SrO–mpg–CN/TiO_2) as efficient heterojunction photocatalysts for the degradation of tetracycline in water. *Adv. Pow. Technol.* **2021,** 32, (8), 2743–2757. https://doi.org/10.1016/j.apt.2021.05.043
2. Liu, G.; Wang, G.; Hu, Z.; Su, Y.; Zhao, L., Ag_2O nanoparticles decorated TiO_2 nanofibers as a p-n heterojunction for enhanced photocatalytic decomposition of RhB under visible light irradiation. *Appl. Surf. Science.* **2019,** 465, 902–910. https://doi.org/10.1016/j.apsusc.2018.09.216
